# Genome-wide identification and characterization of superoxide dismutases in four oyster species reveals functional differentiation in response to biotic and abiotic stress

**DOI:** 10.1186/s12864-022-08610-9

**Published:** 2022-05-18

**Authors:** Youli Liu, Zhenmin Bao, Zhihua Lin, Qinggang Xue

**Affiliations:** 1 Ninghai Institute of Mariculture Breeding and Seed Industry, Zhejiang Wanli University, Ninghai, 315604 China; 2 Zhejiang Key Laboratory of Aquatic Germplasm Resource, Zhejiang Wanli University, Ningbo, 315100 China; 3 College of Marine life Sciences, Ocean University of China, Qingdao, 266100 China

**Keywords:** SOD family, Genomic analysis, Oysters, Diversity

## Abstract

**Background:**

Oysters inhabit in the intertidal zone and may be suffered from environmental stresses, which can increase the production of reactive oxygen species (ROS), resulting in mass mortality. Superoxide dismutases (SODs) protect oysters from ROS damage through different mechanisms compared with vertebrates. However, the molecular and functional differentiation in oyster SODs were rarely analyzed.

**Result:**

In this study, a total of 13, 13, 10, and 8 candidate SODs were identified in the genome of *Crassostrea gigas*, *Crassostrea virginica, Crassostrea hongkongensis*, and *Saccostrea glomerata* respectively. The domain composition, gene structure, subcellular locations, conserved ligands, and cis-elements elucidated the SODs into five groups (Mn-SODs, Cu-only-SODs, Cu/Zn ion ligand Cu/Zn-SOD with enzyme activity, Zn-only-SODs, and no ligand metal ions Cu/Zn-SODs). For single domain Cu/Zn-SODs, only one cytosolic Cu/Zn-SOD (*cg_XM_034479061.1*) may conserve enzymatic activity while most extracellular Cu/Zn-SOD proteins appeared to lose SOD enzyme activity according to conserved ligand amino acid analysis and expression pattern under biotic and abiotic stress in *C. gigas*. Further, multi-domain-SODs were identified and some of them were expressed in response to biotic and abiotic stressors in *C. gigas*. Moreover, the expression patterns of these genes varied in response to different stressors, which may be due to the cis-elements in the gene promoter.

**Conclusion:**

These findings revealed the most extracellular Cu/Zn-SOD proteins appeared to lose SOD enzyme activity in oysters. Further, our study revealed that only one cytosolic Cu/Zn-SOD (*cg_XM_034479061.1*) may conserve enzymatic activity of SOD. Moreover, the expression patterns of these genes varied in response to different stressors, which may be due to the cis-elements in the promoter. This study provides important insights into the mechanisms through which oysters adapt to harsh intertidal conditions, as well as potential biomarkers of stress response in related species.

**Supplementary Information:**

The online version contains supplementary material available at 10.1186/s12864-022-08610-9.

## Background

Superoxide dismutases constitute a group of enzymes that catalyze the conversion of superoxide anion (O^2−^), a hallmark of reactive oxygen species (ROSs), into hydrogen peroxide (H_2_O_2_) and oxygen molecules using a metal cofactor as the catalyst [1–3]. These biochemically similar metalloenzymes are diverse at the genetic and molecular levels. They are categorized into three classes, Cu/Zn-SOD, Fe-SOD/Mn-SOD, and Ni-SOD, and use Cu^2+^, Fe^2+^ or Mn^2+^, and Ni^2+^ as catalytic metal cofactors, respectively [4, 5]. These three classes of SOD likely evolved from three different ancestral genes, giving rise to three distinct protein families with differences in amino acid sequences and molecular conformations. Fe-SOD and Mn-SOD are believed to be phylogenetically related and are thus classified into the same SOD family [6]. Different SODs also vary in their distribution in organism domains and two classes of SOD (Mn-SOD and Cu/Zn-SOD) have been identified in eukaryotes [7, 8]. Additionally, the increased speed of evolution of the Cu/Zn-SODs in the past 100 million years may have further contributed to the molecular variation of the SOD class. In animals, two subclasses of Cu/Zn-SODs, the cytoplasmic Cu/Zn-SOD and the extracellular Cu/Zn-SOD, presumably evolved following independent evolutionary paths and are believed to function the cytosol and mitochondria and in the extracellular milieu, respectively [9–12].

Unlike most vertebrates, where a single molecular form for each of the Mn-SOD, cytosolic Cu/Zn-SOD, and extracellular Cu/Zn-SOD has been identified, some invertebrates appear to have much more complex SOD repertoire [4, 12]. In bivalves, research at the genetic and genomic levels has evidenced the expansion of SOD genes, with identified genes in a species varying from 4 to 10 [13–16]. In addition to their diversity in gene numbers, bivalve SOD genes, particularly those of the Cu/Zn-SOD family, show high variations in the structure and the coded molecules. For example, the glycoprotein pernin isolated from the plasma of the New Zealand green-lipped mussel *Perna canaliculus* and *SOD4*, a potential protein predicted from the scallop *Chlamys farreri*, contain 3 and 4 Cu/Zn-SOD domains, respectively [14]. Moreover, BLAST search-based analysis identified a gene in the Pacific oyster *C. gigas* genome that likely encodes a copper-only SOD-repeat protein (CSRP), which represents a special form of the newly discovered Cu-only SODs [17]. Moreover, the purification and characterization of the most abundant protein in the plasma of 2 oyster species (i.e., Cg-EcSOD or cavortin for *C. gigas* and dominin for the eastern oyster *Crassostrea virginica*) as a homolog of extracellular Cu/Zn-SOD further illustrates the molecular diversity of SODs in bivalves [18–20] Therefore, the molecular diversity of the SOD families and their related evolutionary mechanisms are far from clear for bivalve mollusks.

Living cells produce ROSs during aerobic metabolism [12]. Under physiological conditions, some of these oxidative radicals are pivotal in ROS signaling [1, 21]. However, when organisms are under environmental stress (e.g., pollution, pathogen invasion, abrupt changes in physicochemical conditions), excessive production of ROSs results in oxidative stress, leading to lipid, protein, and DNA damage in the host cells [1]. SODs play an important role in regulating ROS signaling and function as the first line of defense against oxidative damage [3]. The link between SOD malfunction including molecular mutations and a variety of pathological conditions in plants, animals and humans reflects the significance of these proteins for the maintenance of redox homeostasis in different organisms [22–24]. Moreover, recent findings have indicated that the cytosolic SOD1 of yeasts relocates to the nucleus to function as a transcription factor and regulate ROS signaling-related genes. Additionally, SOD1 activates a transcription factor involved in the response to copper deficiency. Collectively, these findings provide preliminary insights into the function of SOD1; however, its mechanisms of action yet to be elucidated [25, 26].

Bivalves often face a wide variety of biotic and abiotic stressors that readily induce oxidative stress owing to the turbulent living environments in estuarine and coastal areas and their filter-feeding lifestyle [14, 27–29]. An increasing body of evidence indicates that the expression of SOD genes changes during stress responses in bivalves such as oysters [15], mussels [30], and clams [31, 32]. However, some homologous proteins of SODs (e.g., *C. gigas* Cg-EcSOD/cavortin and *C. virginica* dominin) do not possess SOD enzyme activity and are thus unlikely to participate in the host antioxidant defense as a direct ROS scavenger [19, 20]. Instead, recent studies have suggested their involvement in host immunity [18, 33] and in metal transportation and storage related processes such as biomineralization [20, 34]. Additionally, the functions and associated mechanisms of action of the multi-SOD domain containing CSRPs remain unknown. Therefore, the functions and regulatory mechanisms of SODs and their homologous proteins in oysters and possibly bivalve mollusks in general are likely more diverse than currently thought, and therefore additional studies are required to bridge these knowledge gaps.

Here, we identified and characterized the SODs of four oyster species (*C. gigas*, *C. virginica, C. hongkongensis*, and *S. glomerata*). Further, genome mapping was conducted, and the gene structure of SODs was characterized in the four aforementioned oyster species. The evolutionary relationship of SODs with other vertebrates and bivalves was also evaluated. Additionally, conserved motif and protein models were also constructed to identify their function. Finally, the cis-element in the promoter and the expression pattern of these SODs under abiotic and biotic stress were used to analyze stress tolerance.

## Results

### Physicochemical characterisitics of predicted oyster sod family members

A total of 44 candidate SOD family genes, 13 in each of *C. giga* and *C. virginica,* 10 in *C. hongkongensis*, and 8 in *S. glomerata*, were identified in the 4 oyster species analyzed in the present research (Table 1). One gene from each oyster species (i.e., *cg_NM_001308918.1* in *C. gigas*, *CH_C06G01251* in *C. hongkongensis*, *cv_XM_022435096.1* in *C. virginica*, and *Sgl008229* in *S. glomerate*) was determined by domain search to contain a Sod_Fe_N (PF00081) domain and were thus identified as Fe/Mn-SOD family genes. All the remaining sequences were found to have 1–4 Sod_Cu (PF00080) domains and were therefore identified as the Cu/Zn-SOD family homologs. Most Cu/Zn-SOD family homologs had one Sod_Cu domain, but 2 sequences (*CH_C02G00935* and *Sgl023942*) contained 2 and 7 sequences (*cg_XM_034474372.1*, *cg_XM_011416304.3*, and *cg_XM_011430838.3* in *C. gigas*, *CH_C03G01680* in *C. hongkongensis*, *cv_XM_022485022.1* and *cv_XM_022462766.1* in *C. virginica*, and *Sgl010356* in *S. glomerate*) contained 4 Sod_Cu domains. Alignments of the amino acid sequences in the predicted SOD domain revealed that the oyster Mn-SODs and Cu/Zn-SODs shared 41.3–62.9% and 20.9–72.0% identity respectively with the correspondent homologous proteins in *H. sapiens*, *M. musculu*, and *D. melanogaster*.

**Table 1:** SOD genes of *C. gigas*, *C. virginica*, *C. hongkongensis*, and *Saccostrea glomerata*

ID	Length (aa)	pI	MW (kDa)	Instability index	Aliphatic index	GRAVY
cg_NM_001308888.2	192	5.04	21,253.21	39.2	75.26	−0.493
cg_NM_001308918.1	225	6.54	25,139.87	29.76	94.13	−0.159
cg_XM_011416093.3	192	4.91	21,242.21	39.96	76.77	−0.448
cg_XM_011416094.3	427	5.55	48,390.17	38.1	79.13	−0.357
cg_XM_011416304.3	1022	5.63	109,649.28	38.52	76.1	−0.165
cg_XM_011430838.3	913	7.33	101,412.51	40.12	84.39	−0.252
cg_XM_011436741.3	524	8.44	53,978.29	33.39	43.03	−0.704
cg_XM_011436744.3	335	6.36	34,656.26	31.55	61.1	−0.403
cg_XM_011441495.3	193	5.18	21,288.33	43.78	74.45	−0.438
cg_XM_011448476.3	248	6.12	26,482.05	42.16	90.28	−0.229
cg_XM_034462555.1	227	6.25	25,547.32	37.74	74.32	−0.488
cg_XM_034474372.1	991	5.64	106,399.4	38.84	74.91	−0.201
cg_XM_034479061.1	156	5.84	15,913.73	21.08	77.5	−0.209
cv_XM_022435096.1	226	6.49	25,221.92	29.15	88.54	−0.18
cv_XM_022449081.1	224	6.22	25,564.25	63.78	60.45	−0.864
cv_XM_022449088.1	127	6.04	14,662	72.39	46.85	−1.131
cv_XM_022451521.1	216	5.28	24,000.59	49.41	82.69	−0.358
cv_XM_022451522.1	192	5.04	21,013.03	43.04	76.35	−0.432
cv_XM_022451523.1	192	5.17	20,772.81	43.66	78.91	−0.318
cv_XM_022452516.1	181	6.14	20,429.57	63.41	62.98	−0.754
cv_XM_022452520.1	186	6.32	20,684.45	43.58	79.09	−0.252
cv_XM_022452581.1	174	5.05	19,207.93	39.52	71.32	−0.573
cv_XM_022460203.1	348	6.35	36,093.87	32.15	59.11	−0.433
cv_XM_022462766.1	902	8.39	100,580.69	40.25	79.8	−0.326
cv_XM_022476413.1	251	6.11	26,732.39	38.06	91.99	−0.217
cv_XM_022485022.1	1020	6.2	110,135.22	46.46	78.16	−0.212
CH_C02G00935	341	8.92	38,375.62	36.7	80.32	−0.284
CH_C02G02366	427	5.55	48,442.16	40.47	79.81	−0.363
CH_C02G02368	173	4.73	19,173.9	40.92	72.2	−0.51
CH_C02G02367	192	5.02	21,210.21	37.48	78.8	−0.421
CH_C02G02369	195	4.89	21,809.97	30.37	81.54	−0.416
CH_C03G01680	985	5.8	105,518.42	38.07	74.5	−0.203
CH_C04G01146	248	6.44	26,496.12	40.86	90.28	−0.23
CH_C06G00439	156	5.84	15,925.74	22.32	76.86	−0.231
CH_C06G01251	256	6.66	29,019.22	41.62	81.95	−0.289
CH_C08G01508	1253	7.25	135,387.91	39.44	66.29	−0.449
Sgl008229	224	6.54	24,940.56	28.06	85.89	−0.163
Sgl010356	1019	5.81	110,756.56	42.04	73.61	−0.253
Sgl011055	545	9.19	55,945.35	40.94	41.54	−0.741
Sgl011058	328	6.35	34,108.88	35.71	65.7	−0.312
Sgl016219	252	7.55	27,061.88	36.8	93.57	−0.212
Sgl021124	100	6.11	10,295.46	19.53	80	−0.29
Sgl023942	464	5.45	52,411.63	39.61	67.2	−0.75
Sgl023943	214	5.44	23,492.06	41.62	76.17	−0.361

The proteins encoded by the identified oyster SOD family genes varied greatly in molecular characteristics (Table 1). The 4 Mn-SOD homologs were predicted to have a polypeptide length varying from 224 to 256 aa, with a calculated molecular weight of 24.9–29.0 kDa and an isoelectric point (pI) of 6.49–6.66. The single domain Cu/Zn-SOD family members ranged from 100 aa to 545 aa long, with a calculated molecular weight of 10.3–55.9 kDa and a pI of 4.73–9.19, except one large molecular protein encoded by CH_C08G01508. The 2 and 4 Sod_Cu domain molecules were significantly larger than the 1 domain containing ones, with the 2 two-domain proteins having a molecular weight of 38.4 kDa and 52.4 kDa and the 7 four-domain proteins being 100.6–110.8 kDa.

### Genome mapping, gene duplication and Synteny analysis of SOD family members

**Fig. 1:**
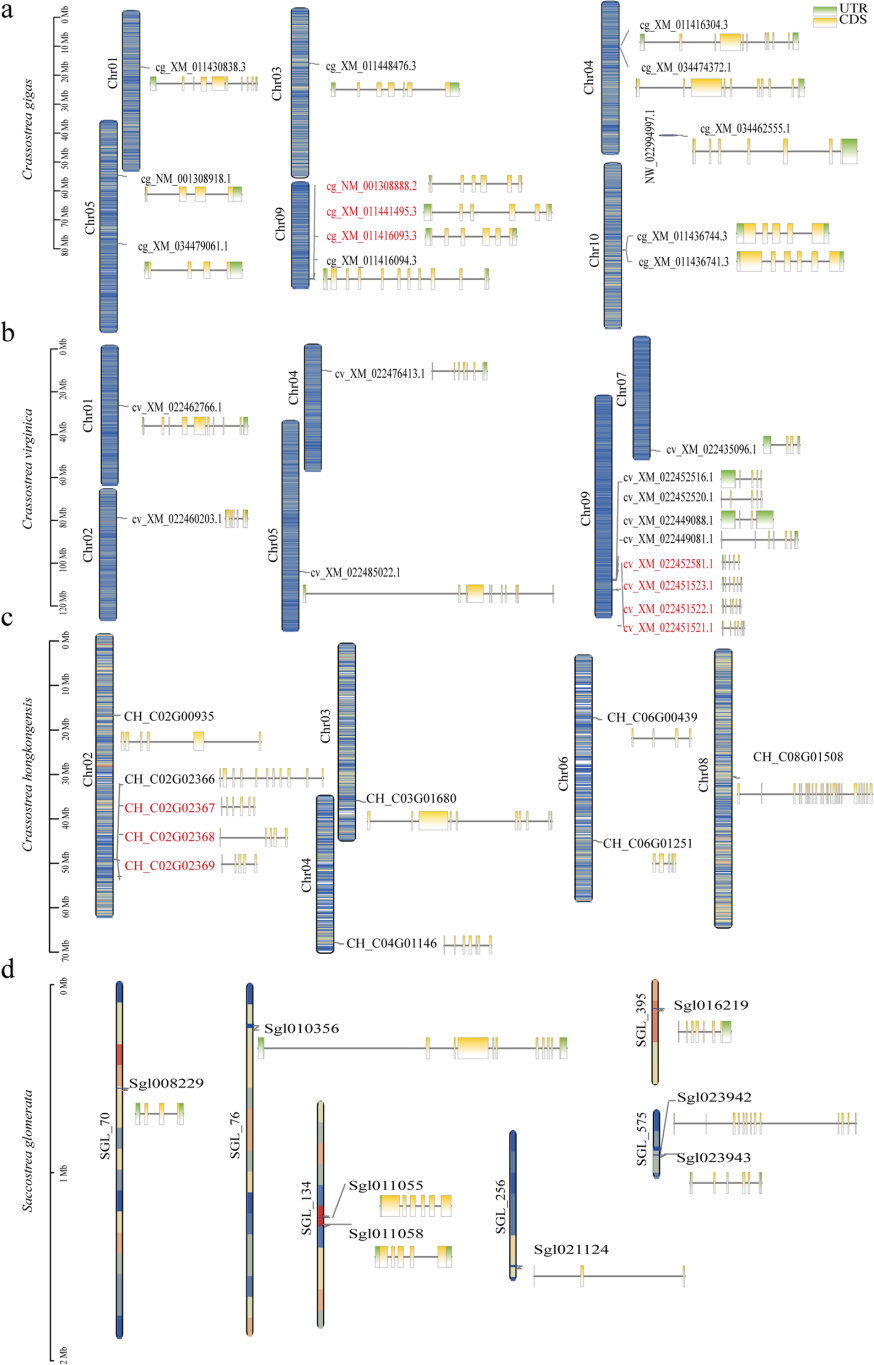
Genome mapping and exon-intron characterization of four oyster species. **a**, **b**, **c**, and **d** Genome location and gene structure of SODs in *Crassostrea gigas*, *Crassostrea virginica, Crassostrea hongkongensis*, and *Saccostrea glomerata*, respectively. The columns represent the chromosome of each oyster, the blue or yellow columns represent the gene density of each chromosome (**a**, **b**, **c**) or scaffold (**d**). The gene structure is indicated in green (UTRs), yellow (CDSs), and black lines (introns). The tandem duplicated genes in each species were colored in red

**Fig. 2:**
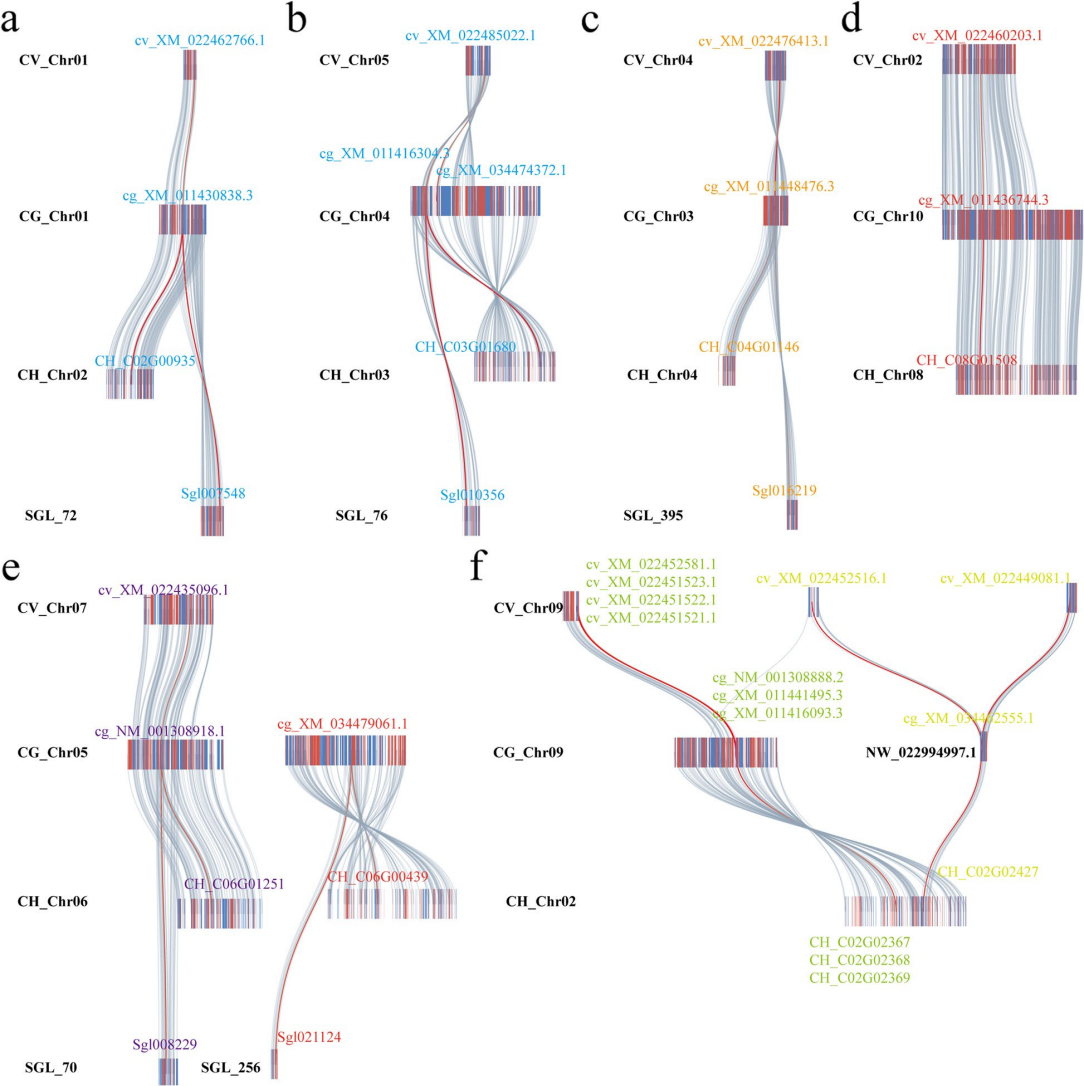
Collinear relationship of SODs in *Crassostrea gigas*, *Crassostrea virginica, Crassostrea hongkongensis*, and *Saccostrea glomerata*. “CG_”, “CV_”, “CH_”, and “SGL_” represented *Crassostrea gigas*, *Crassostrea virginica, Crassostrea hongkongensis*, and *Saccostrea glomerata*, respectively. The red line between chromosome represented the collinear relationship of SOD genes. The same color showed the collinear genes

TBtools mapped the SOD family genes on 6 chromosomes in *C. gigas* and *C. virginica*, 5 chromosomes in *C. hongkongensis* genes, and 6 scaffolds in *S. glomerata* (Fig. 1). Some Cu/Zn-SOD genes were present in a cluster on the same chromosome, with a chromosome of *C. gigas* and *C. hongkongensis* containing 3 tandem duplicated genes and a chromosome of *C. virginica* containing 4 tandem duplicated genes (Additional file 1: Table S1). The structural analysis predicted 4 exons in the Mn-SOD gene of *C. gigas* and *C. virginica*, and *S. glomerata* and 5 exons in that of *C. hongkongensis*. In contrast, the predicted exon numbers of the Cu/Zn-SOD family genes varied between 4 and 11. The synteny result revealed that those SODs were in 6 collinear regions in four oyster species (Fig. 2 and Additional file 2: Fig. S1). The genes in the collinear region showed high similarity in four species. The Cu/Zn-SOD genes (*cg_XM_034479061.1*, *CH_C06G00439*, and *Sgl021124*) have more than 70% similarity with human SOD1 and in the same collinear region (Additional file 3: Table S2). The collinear analysis also showed that the Mn-SODs were conserved in four oyster species. In addition, the tandem duplicated genes of three oysters were in the collinear regions among four species.

### Phylogenetic tree, sequence analysis and enzyme activity prediction

**Fig. 3:**
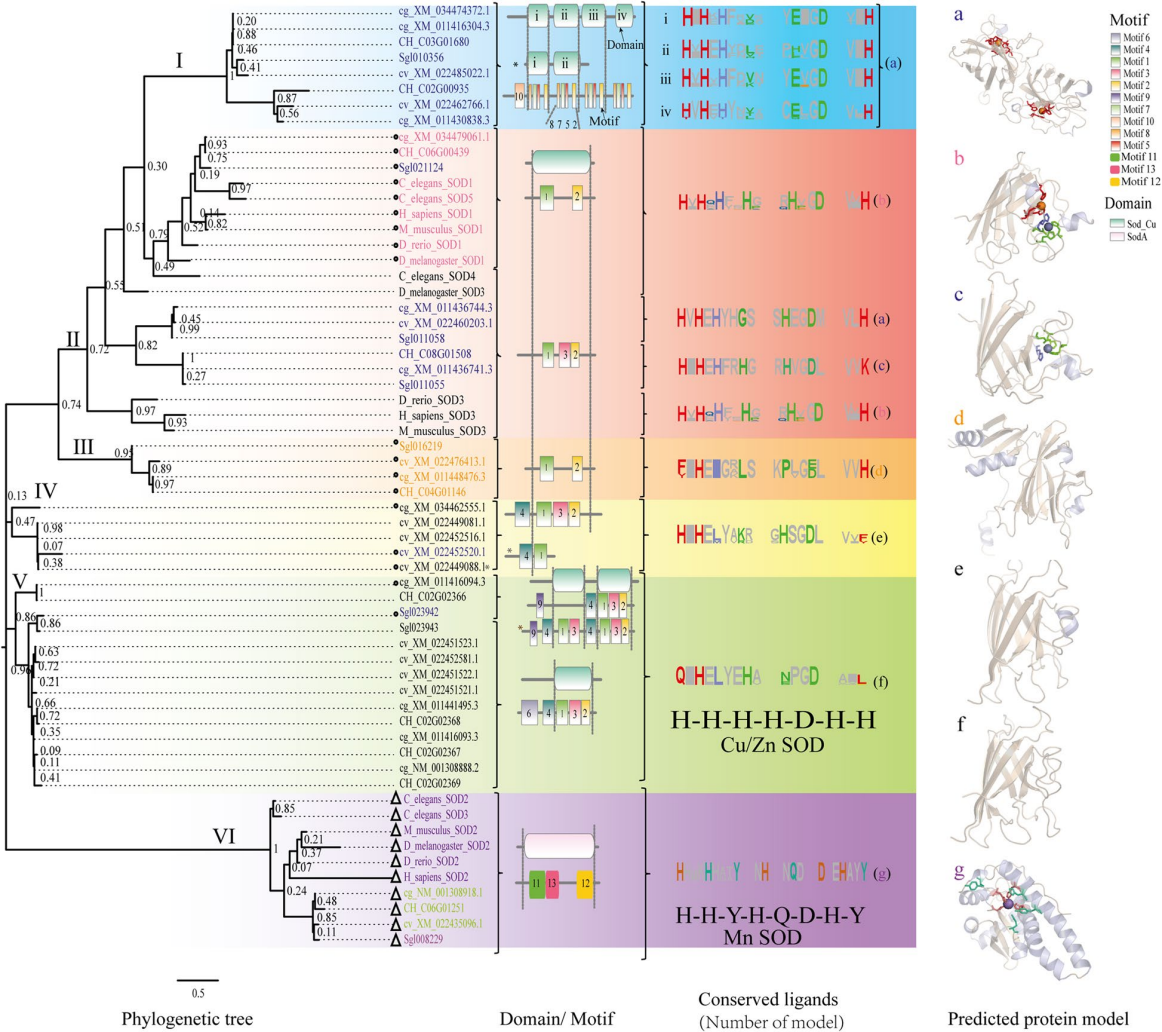
Phylogenetic, Motif/domain, conserved ligands, and protein model analysis of SODs revealed the function diversity. cg: *Crassostrea gigas*, cv: *Crassostrea virginica*, CH: *Crassostrea hongkongensis*, Sgl: *Saccostrea glomerata*, H_sapiens: *Homo sapiens*, M_musculus: *Mus musculus*, D_rerio: *Danio rerio*, D_melanogaster: *Drosophila melanogaster*, and C_elegans: *Caenorhabditis elegans. *“I” to “VI” represented the six clades. Circles, triangles, and no mark near the tree represented the cytosolic, mitochondrial, and extracellular sub-cellular of SODs, respectively. The proteins in seven colors represented the protein models predicted by phyre2. In the Domain/motif part, colored cylinders (green and pink) represent the Cu/Zn-SOD and Mn-SOD domains. The rectangles numbered one to thirteen represent motifs. The Conserved Ligands part displayed the most important ligands for enzyme activity of SODs. The “i”,“ii”,“iii”, and “iv”in both second and third part were the numeric of multi-domain Cu/Zn-SOD (clade I). “a” to “g” in the third and fourth part represented the corresponding models of encoded proteins. In the protein model part, the models were predicted by SWISS-MODEL based on the model predicted in phyre2, so, the same color of the letters in the first, third, and fourth part were same. The orange sphere in “a” and “b” was the copper ion, the blue sphere in “b” and “c” was zinc ion, the purple sphere in “g” was manganese ion

A phylogenetic tree was constructed with the amino acid sequences of SOD family proteins from the 4 oyster species and that from the 5 model animals (*Homo sapiens*, *Mus musculus*, *Danio rerio*, *Drosophila melanogaster*, and *Caenorhabditis elegans*) (Fig. 3). The molecules were grouped into 6 clades according to the phylogenetic and sequence analysis. Clade I, shadowed in light blue, encompassed 7 four-domain and 1 two-domain Cu/Zn-SOD homologs and all the coded proteins were predicted to distribute extracellular (Additional file 4: Table S3). Clade II, shadowed in watermelon red, included 9 single domain Cu/Zn-SODs that were predicted to distribute in cytoplasm (cyto-Cu/Zn-SODs) and 11 in extracellular milieu (ec-Cu/Zn-SODs). Clade III, shadowed in orange, included 4 cyto-Cu/Zn-SODs (95% bootstrap value), had different predicted protein model with other clades. Clade IV, shadowed in yellow, included the cyto- and ec-Cu/Zn-SODs. Clade V (86% bootstrap value), shadowed in green, Cu/Zn-SODs also included the cyto- and ec-members. Clade VI (100% bootstrap value), shadowed in light purple, included all the Mn-SOD molecules.

The MEME server predicted 10 conserved motifs (numbered 1 to 10) in the Cu/Zn-SOD sequences (Additional file 5: Table S4) and 3 motifs in the Mn-SOD sequences. Motif 1 (DHNHGLQIHEYGDMEHGCDTIGELYHNEH) contained six of the Cu/Zn-SOD conserved ligand site region and motif 2 contained the fourth Cu ligand histidine. Motif 1 and 2 also corresponded to the domain Sod_Cu (PF00080) and represented the Cu/Zn-SOD superfamily. In addition, motifs 8, 7, 2 contained two, one, one of the four Cu ligands, respectively. In Clade I, each Cu/Zn-SOD domains contained the combination motif 8–7–5-2. In Clade II, two kinds of combination of motif 1–2 and 1–3-2 represented the Cu/Zn-SOD domain. In Clade III, the 1–2 motif combination was identified. In Clade IV and Clade V, the common motif combination was 1–3-2. In addition, the 1–3-2 motif was the most frequent among all combinations in all the Cu/Zn-SODs. In Clade VI, the motif 11 to 13 covered the sodA#PF00081. All the oysters’ sequences were similar in the predicted motif patterns to the family representative molecule from the model species.

Amino acid sequence alignments of the oyster SOD homologs with that of the representative molecules from model species identified variable number of conserved amino acid residues that assumingly served as metal ion ligands (CLs) (Fig. 3 conserved ligands). For family Cu/Zn-SODs, 7 residues are determined to serve as the CLs for the ions Cu and Zn in the enzyme active center and thus be essential for the SOD enzyme activity in known Cu/Zn-SODs [4, 8, 9]. In the 4-domain sequences in Clade I, each domain possessed 3–6 CLs (Additional file 6: Table S5). The sequences in Clade II had 6 (b and c) or 7 (a) CLs. The polypeptides encoded by *cg_XM_034479061.1*, *cg_XM_011436744.3*, *CH_C06G00439*, *cv_XM_022460203.1*, *Sgl021124*, and *Sgl011058* in this clade contained all the 4 Cu CLs and 2 or 3 of the Zn CLs. The CL numbers in the sequences grouped in Clades III-V varied between 2 and 4, with the partial loss of ligands for both Cu and Zn. For the Mn-SOD homologous sequences in Clade VI, all the 8 CLs, His-His-Asp-His for Mn^2+^ linkage and His-Tyr-Gln for maintaining the active center confirmation [5], were conserved.

Structure modeling of all the oyster SOD homologous proteins with Phyre2 identified 7 most accurate structure models corresponding to the predicted molecules different in motif and CL compositions (Fig. 3 predicted protein models a-g, Additional file 7: Table S6 by phyre2, Additional file 8: Table S7 by SWISS-MODEL). The structural model developed on the polypeptides in Clade I showed a SOD active center that contained a copper ion, but no zinc ion (model a). The predicted structure of proteins in Clade II involved 3 models that showed an active center containing a copper ion (model a), both copper and zinc ions (model b), or a zinc ion (model c). The proteins in Clades III, IV and V were developed into another 3 models (models d, e, f) that all lacked a metal ion containing active center. The model a-f also had a different number of α helix and coil/β-turns while they shared some structural elements in particular the eight β strands. The existed of eight β strands may contribute to the similar structure between models a-f. The CLs meanly existed on the α helix or coil/β-turns, may cause the loss of ligand metals. However, the structure model of the proteins in Clade VI developed based on 2 known Fe/Mn-SODs (Fe-SOD and Mn-SOD are same protein only varied in ligand ions) differed significantly in confirmation from the other with the other models and showed an active center that contained a manganese ion (model g).

### Cis- regulatory elements in the regulatory region of sod genes

**Fig. 4:**
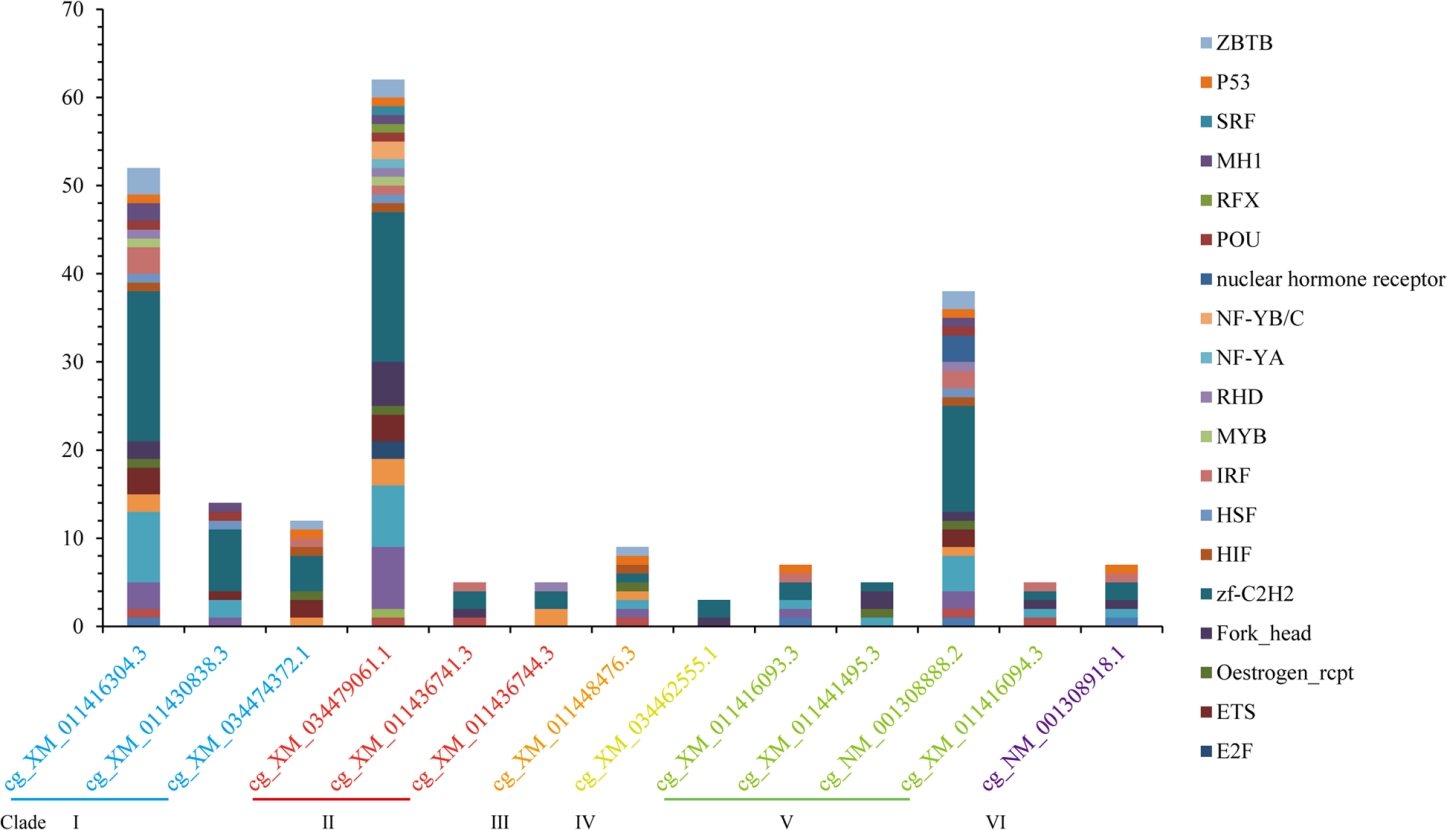
Statistics of the TF families of *Crassostrea gigas* SODs. The x-axis showed the SODs of *Crassostrea gigas*, the y-axis showed the number of transcription factor families. The SODs were marked into six colors from clade “I” to “VI”

A total of 105 transcription factors (TFs) involving 25 families as the cis-elements were identified in the promoter of SODs in the Pacific oyster (Fig. 4 and Additional file 9: Table S8). Androgen receptor represented the most frequently identified TF in all the Pacific oyster SOD genes. Approximately 30% of the TFs involving 14 TF families were unique to the oyster SODs. Among them, the family Forkhead (FOX) was the most frequently identified. These unique TFs were present in the Mn-SOD gene (*cg_NM_001308918.1*), and some Cu/Zn-SOD family genes (Clade I, *cg_XM_011416304.3*; Clade II, *cg_XM_034479061.1*, *cg_XM_011436744.3*, Clade IV *cg_XM_034462555.1*, and Clade V, *cg_NM_001308888.2*, *cg_XM_011441495.3*).

The numbers of cis-element identified in individual genes varied between 2 and 6 (Additional file 10: Table S9). The promoter of *cg_NM_001308918.1*(Mn-SOD) and *cg_XM_011416304.3*, for example, contained 6 cis-elements, while *cg_XM_011430838.3* only had 2 cis-elements. Most genes were identified to have 3–5 cis-elements in their promoter.

### SOD expression profiles in development stage and adult pacific oyster tissues

**Fig. 5:**
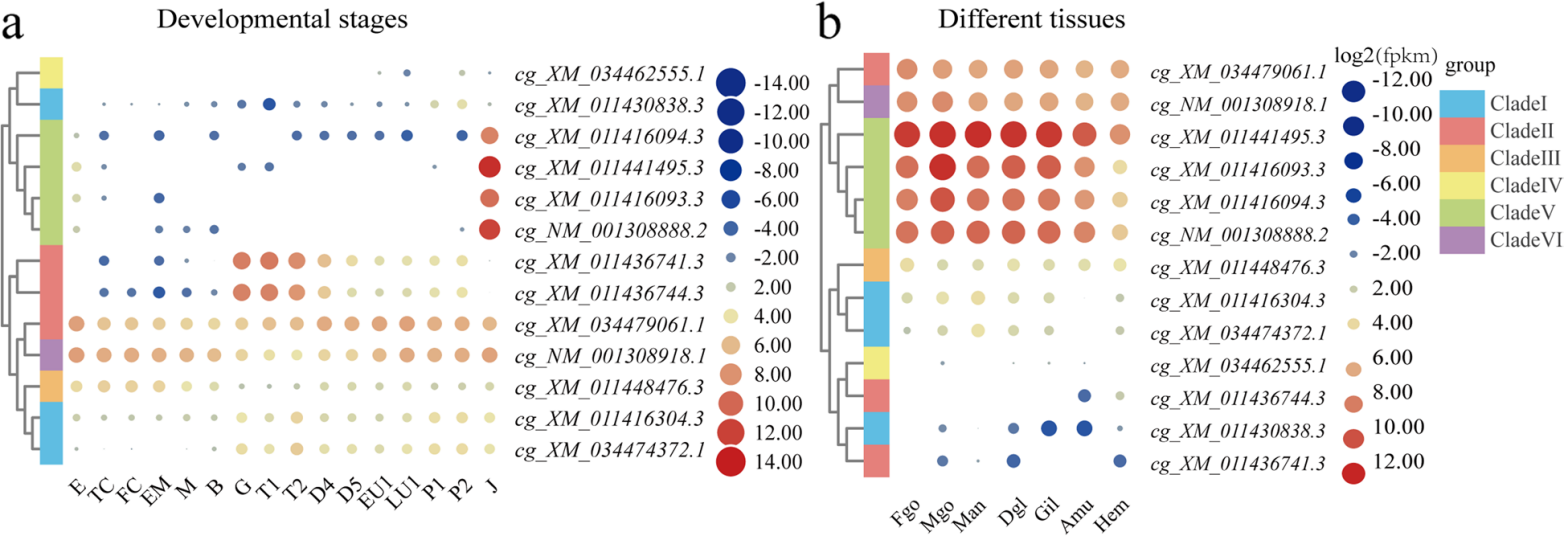
Heat map of *Crassostrea gigas* SOD expression at different developmental stages and in different tissues. The legend represented the log2 transformation for expression level of each SOD. **a** Heat map of *Crassostrea gigas* SOD expression during development. E: egg, TC: two cells, FC: four cells, EM: early morula, M: morula, B: blastula, G: gastrula, T1/T2: trochophore1/2 (9.5 h/11.5 h), D4/D5: D-shape larvae 4/5 (20.5 h/1.09 d), EU1: early-umbo larvae (4.77 d), LU1: late-term umbo larvae (14.73 d), P1/P2: pediveliger 1/2 (18.03 d/18.19 d), J: juvenile. **b** Heat map of *Crassostrea gigas* SOD expression in different tissues. Fgo: Female gonad, Mgo: male gonad, Man: mantle, Dgl: digestive gland, Gil: gill, Amu: adductor muscle, Hem: hemocyte

During embryonic and larval development, the expression patterns varied among SOD genes (Fig. 5a). A set of genes, including two cyto-Cu/Zn-SODs (seven CLs, *cg_XM_034479061.1*, two CLs, *cg_XM_011448476.3*), a multi-domain-SOD (*cg_XM_011416304.3*), and Mn-SOD, were detected in all developmental stages. The expression level of two ec-Cu/Zn-SODs that contained 6 and 7 CLs exhibited an increasing trend from the gastrula stage. A cyto-Cu/Zn-SOD and three ec-Cu/Zn-SOD that contained three CLs expressed an extremely high level during the juvenile stage but almost not expressed in other stages (Fig. 5a). Additionally, those four genes were the most highly expressed among all SOD genes in adult tissues (Fig. 5b). The cyto-Cu/Zn-SOD containing seven CLs and Mn-SOD showed higher mRNA level in all tissues and with similar expression levels among different tissues.

### Expression pattern of SOD genes under abiotic and biotic stress

**Fig. 6:**
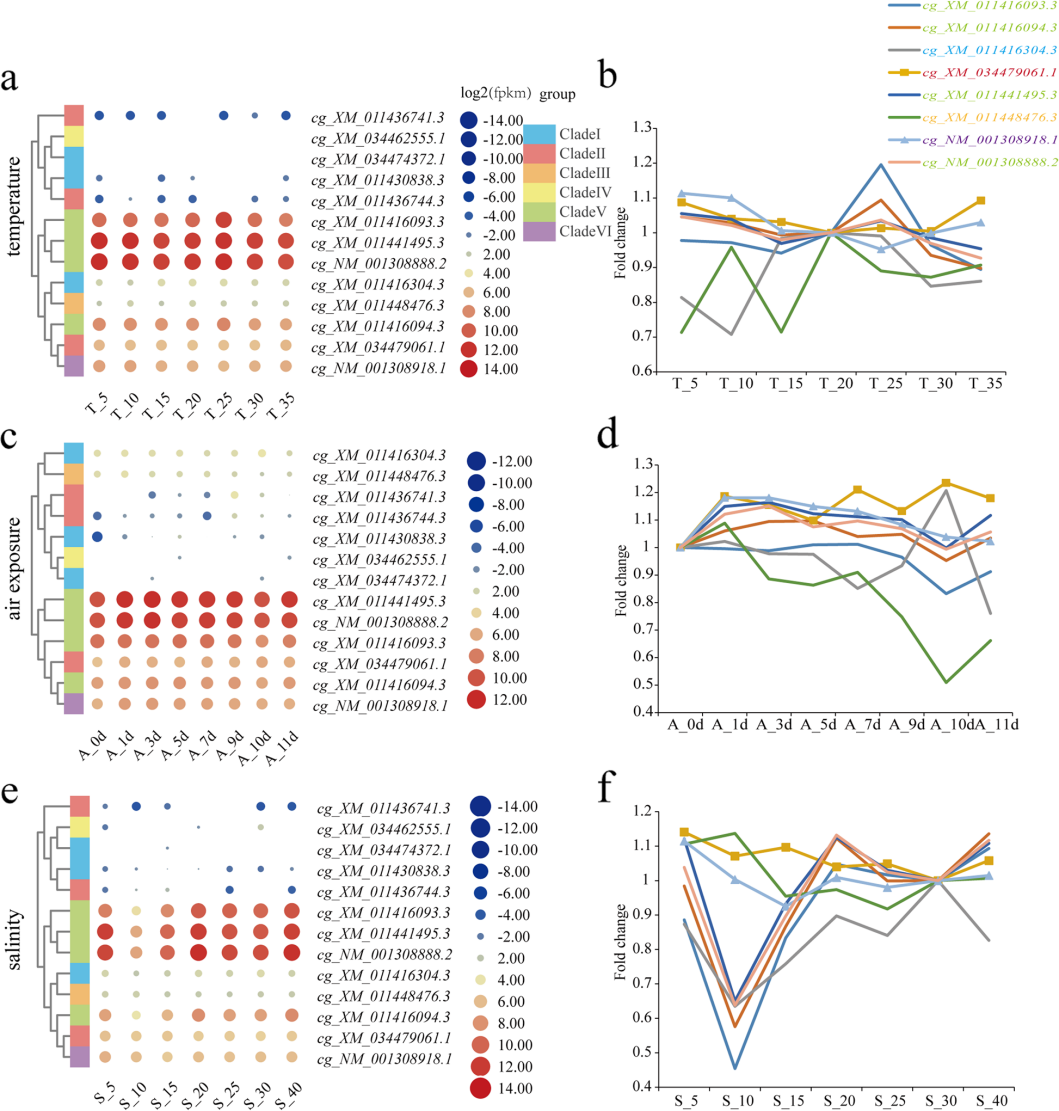
Heat map and line chart of *Crassostrea gigas* SOD expression in response to abiotic stress. The legend represented the log2 transformation for expression level of each SOD. **a**, **b** Heat map of *Crassostrea gigas* SOD expression in response to temperature stress. T_5, T_10, T_15, T_20, T_25, T_30, T_35, represent stress on 5, 10,15, 20, 25, 30, and 35 °C, respectively. **c**, **d** Heat map of *Crassostrea gigas* SOD expression in response to air exposure stress. A_0d, A_1d, A_3d, A_5d, A_7d, A_9d, A_10d, A_11d, represent air exposure for 0, 1, 3, 5, 7, 9, 10, and 11 days, respectively. **e**, **f** Heat map of *Crassostrea gigas* SOD expression in response to salinity stress. S_5, S_10, S_15, S_20, S_25, S_30, S_40, represent salinities of 5, 10, 15, 20, 25, 30, and 40 psu, respectively. **b**, **d**, **f** Fold change of SODs in response to the aforementioned stress conditions

We further investigated the transcriptional profiles of SOD genes in adult Pacific oysters subjected to abiotic and biotic stress. The genes in Clade V (*cg_XM_011416093.3*, *cg_XM_011416094.3*, *cg_XM_011441495.3*, and *cg_NM_001308888.2*) were highly expressed and up-regulated at 25 °C under different levels of temperature stress (Fig. 6a,b). The Mn-SOD and cyto-Cu/Zn-SOD that contained seven and two CLs (*cg_XM_034479061.1* and *cg_XM_011416094.3*) were up-regulated under air exposure stress (Fig. 6c,d). Under low salinity (salinity 5, 10 psu), six SODs were down-regulated, whereas the cyto-Cu/Zn-SOD that contained seven CLs and Mn-SOD were up-regulated in eight responsive genes (Fig. 6e,f).

**Fig. 7:**
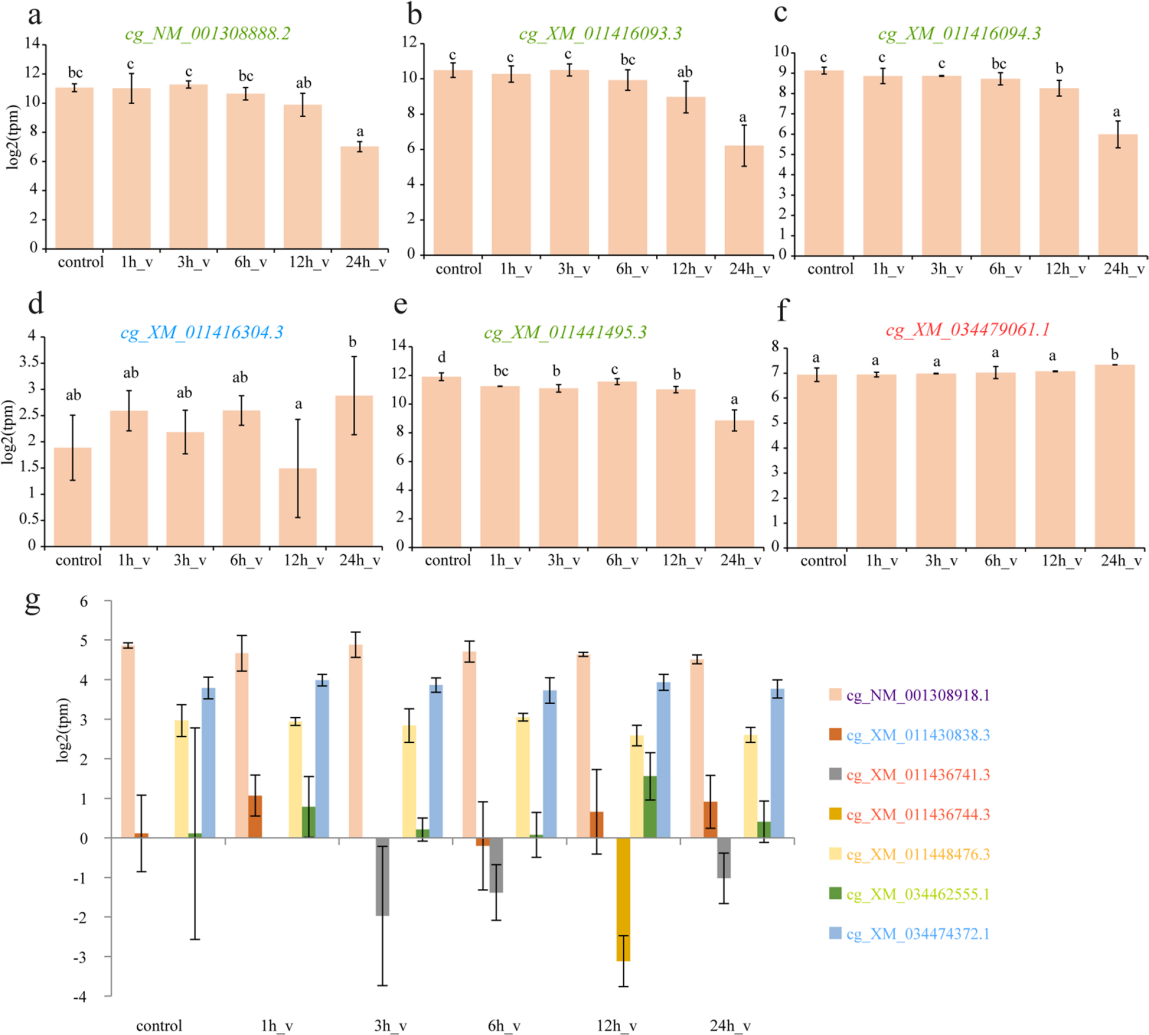
SOD expression patterns in *Vibrio* challenged *Crassostrea gigas*. Expression pattern of (**a**) *cg_NM_001308888.2*, (**b**) *cg_XM_011416093.3*, (**c**) *cg_XM_011416094.3*, (**d**) *cg_XM_011416304.3*, (**e**) *cg_XM_011441495.3*, and (**f**) *cg_XM_034479061.1*. (**g**) Expression pattern of SODs without significant change under *Vibrio* stress. Significant differences were identified via one-way ANOVA followed by Duncan’s test (*P* < 0.05)

In response to biotic stress, six genes (including four SODs in Clade V, one multi-domain-SOD in Clade I (*cg_XM_011416304.3*), and one cyto-Cu/Zn-SOD in Clade II contained seven CLs) exhibited significant changes (*P* ≤ 0.05) with temporal activation (Fig. 7), whereas the mRNA level of other genes showed no differences (*P* > 0.05) during infection. The four SODs in Clade V exhibited the same trends, thus demonstrating that these genes were down-regulated in response to infection, while the multi-domain-SOD (*cg_XM_011416304.3*) and cyto-Cu/Zn-SOD containing seven CLs were up-regulated during *Vibrio* infection.

## Discussion

Our findings indicated that the SOD family genes expanded in four oyster species (*C. gigas*, *C. virginica, C. hongkongensis*, and *S. glomerata*) compared with those of vertebrates [35, 36]. ec-SODs represent the majority of Cu/Zn-SODs, which is consistent with that the ec-SOD evolutionary history is longer than that of cytoplasmic Cu/Zn-SODs [9]. The SODs were clustered into six clades according to the phylogenetic analysis. The Mn-SODs of the four oyster species had two kinds of protein models, which were predicted to be different chains of iron-SOD [37]. Our results thus confirmed that Mn-SODs are much conserved than Cu/Zn-SODs [38].

For the Cu/Zn-SODs, the multi-domain-SODs and single-domain SODs were first separated. The predicted protein model of multi-domain-SODs in clade I was heterodimers of the mutant SOD enzyme, which had been reported in previous studies. In *Chlamys farreri*, one multi-domain-SOD was identified, whereas two to three genes were identified in the three *Crassostrea* oysters [14]. Multi-domain-SODs have only been identified in several aquatic organisms and winged insects [17], however, their function is not characterized yet. In the present study, we found that the predicted protein model showed they were Cu-only SODs and might have the SOD enzyme activity. In the expression pattern study, a multi-domain-SOD (*cg_XM_011430838.3*) expressed lowest level in different tissues and under abiotic and biotic stress. Another two multi-domain SODs (*cg_XM_011416304.3* and *cg_XM_034474372.1*) were down-regulated under abiotic and biotic stress (Figs. 5 and 6). The mRNA expression of Cu-only SOD (multi-domain-SOD in this study) in the Pacific oyster decreased significantly in gills exposed to Cu but increased under Pb exposure [39]. The function of multi-domain-SODs in fungi was related to defense host-derived oxidative stress [40]. The function of oyster multi-domain-SODs is still unclear, and therefore their functions and expression patterns should be further characterized.

Except for the Mn-SOD and multi-domain-SODs, single domain SODs were separated into four subgroups according to the number of CLs. Cyto-Cu/Zn-SODs contained seven CLs, among which *cg_XM_034479061.1* and *CH_C06G00439* were predicted to have the same structure as human SOD1 [41] and were more likely to possess enzyme activity [9]. The protein structures of ec-Cu/Zn-SODs, which contained two CLs (clade V), had been purified and identified from *C. virginica* [20] and *C. gigas* [19]. The studies revealed that these ec-Cu/Zn-SODs (major plasma proteins) possessed the features of the Cu/Zn-SOD domain but lacked SOD enzyme activity [20]. Thus, the cyto-Cu/Zn-SODs that contained two CLs (*cg_XM_034462555.1*, and *cg_XM_011448476.3*) might lack SOD enzyme activity similar to the ec-Cu/Zn-SODs that contained two CLs (*cg_XM_011441495.3*, *cg_NM_001308888.2*, and *cg_XM_011416093.3*). In the present study, the recombinant protein of *cg_XM_034479061.1* was proved to have SOD activity, and the loss of Cu CLs in *cg_XM_034479061.1* caused the SOD activity could not be detected [42]. The result proved the importance of Cu ligands. The predicted cyto-Cu/Zn-SOD (*cg_XM_011416094.3*) was a ec-Cu/Zn-SOD in the previous study and lack SOD enzyme activity [43]. Although the SOD1 mutant of the CLs might cause enzyme activity loss and gain other kinds of function in humans [24, 44], Cu/Zn-SODs with no enzyme activity may participate in the oxidation defense by transporting metal [20, 34, 39, 43]. Additionally, those oyster cyto-Cu/Zn-SODs contained three or two CLs were predicted as the heterodimer between the copper chaperone for SOD and SOD1, which may facilitate or regulate copper delivery [45]. Thus, the SODs that contained two CLs might provide different protective functions compared with SOD1 in humans. The cyto-Cu/Zn-SOD that contained seven CLs was up-regulated, whereas the ec-Cu/Zn-SOD that contained six CLs (all Cu CLs remained) *cg_XM_011436744.3* was not significantly up-regulated in response to biotic and abiotic stress. Those result suggested that the only single domain Cu-only gene (*cg_XM_011436744.3*) might be a pseudogene or nearly no function by no expression in the adult organisms. All of the results revealed that the cyto-Cu/Zn-SOD with seven CLs (*cg_XM_034479061.1*) might be the only cyto-Cu/Zn-SOD with SOD enzyme activity in *C. gigas*. For other ec-Cu/Zn-SODs in *C. gigas*, 4 (Clade V) in 6 were proved with no SOD enzyme activity [20, 42]. Another ec-Cu/Zn-SOD (*cg_XM_011436744.3*, Clade II,) was predicted to lost 1 of 4 Cu ligands, might lack the SOD activity according to previous study [42, 46]. Thus, except for the multi-domain Cu/Zn-SODs, there might be no or only one (*cg_XM_011436744.3*) ec-Cu/Zn-SOD play the role with SOD activity in *C. gigas*. Although all the Cu/Zn-SOD possessed the SOD-domain, variable functions have been identified in previous studies [20, 34, 40], and therefore the presence of domains is not enough to indicate the function of genes, the conservation of key functional sites and other aspects to facilitate need to be considered in future analyses. Altogether, our findings indicate that SODs in oysters may be categorized into five groups according to their functional diversity (Mn-SOD enzyme, Cu-only-SODs, Cu/Zn ion ligand Cu/Zn-SOD with enzyme activity, Zn-only-SODs, and no ligand metal ions Cu/Zn-SOD); however, the relationship between different functions requires further characterization.

We further studied the cis-element and expression patterns of *C. gigas* SODs due to their wide variations. The androgen receptor (AR) was present in most SODs. It had widespread expression in many cells and tissues, and the transcriptional regulatory effects of the AR included pathways involved in cell growth and proliferation, cell cycle progression, protein synthesis, and cell death [47]. Estrogen receptor 1 (ESR1) was found in the promoter of six Cu/Zn-SODs. AR and ESR1 are members of the steroid hormone nuclear receptor family, which was involved in a wide variety of physiological functions, including control of embryonic development, cell differentiation, and homeostasis [48]. The chromosome remodeling factor family members are key components involved in DNA replication and include histone chaperones, histone-modifying enzymes, and ATP-dependent chromatin remodeling complexes. The ec-Cu/Zn-SOD (*cg_NM_001308888.2*) had the most TFs compared with the other two ec-Cu/Zn-SODs in clade V. All of these ec-Cu/Zn-SODs were not up-regulated at low or high temperatures (35 °C). These ec-Cu/Zn-SODs were distributed in clusters on one chromosome, which was consistent with a previous study that the Pacific oyster was enriched with duplication regions [49]. The duplication of genes might still share transcriptional characteristics as demonstrated by the expression patterns under abiotic and biotic stress. The similar expression profile might due to the preservation of the promoter of duplicated genes [50]. A previous study showed that the Cu/Zn-SODs that contained two CLs in Clade V (*cg_XM_011416093.3*, *cg_XM_011416094.3*, *cg_XM_011441495.3*, and *cg_NM_001308888.2*) were up-regulated in response to metal-induced stress, suggesting that they might participate in metal detoxification [39]. In the absence of CLs, oxidative defense mechanisms may lose, for instance, human mutant SOD1 lacked the Cu or Zn ligand caused the SOD1 malfunction, which leads to loss of SOD activity [23, 41]. The genes in the same clade shared some similar TFs, that is critical for their response to the same condition. In addition, unique TFs of genes in Clade V may contribute to their metal detoxification (sub-functionalization process) [50, 51]. Therefore, more studies are needed to analyze the diversity of the cis-elements in promoters to gain insights into the functional evolution of genes. The diversity of cis-elements in the promoter revealed the potential regulatory relationship between different cell processes [52]. Our work lays the foundation for future comparative studies to elucidate the functional diversity of SOD family and underlying mechanisms.

## Conclusions

SOD genes were analyzed in the genomes of four oyster species. Cu/Zn-SOD exhibited a wide diversity of gene family numbers and sub-cellular localization, chromosomal distribution, gene structure, and conserved ligand amino acids (CLs), whereas these features were highly conserved for Mn-SOD. Most extracellular Cu/Zn-SOD proteins appeared to lose SOD enzyme activity in oysters due to the lack of CLs despite the occurrence of more than one gene and higher levels of gene expression. Therefore, the functions of this kind of Cu/Zn-SOD must be further characterized. Multi-domain-SODs are widely distributed in oysters and some of them responded to biotic and abiotic stressors in the Pacific oyster. Further, our study revealed that only one cytosolic Cu/Zn-SOD (*cg_XM_034479061.1*) may conserve enzymatic activity of SOD. Moreover, the expression patterns of these genes varied in response to different stressors, which may be due to the cis-elements in the promoter. Our results clarified the gene family members and potential functions of SODs in oysters, as well as the unusual diversity of SODs. These findings provide important insights into the mechanisms by which oysters adapt to harsh intertidal conditions and allows for the identification of precise biomarkers of stress response in oysters and other invertebrates.

## Methods

### Identification, sequence and structure analyses of SOD family members

The initial chromosome/scaffold sequences were downloaded from a public online database, including *C. gigas* (assembly cgigas_uk_roslin_v1, GCA_902806645.1) [53] and GCA_002022765.4 C_virginica-3.0 on NCBI, *C. hongkongensis* genome [54] at the China National GenBank (CNGB, https://db.cngb.org/, No. CNA0003280), as well as the Sydney rock oyster *S. glomerata* genome online database (http://soft.bioinfo-minzhao.org/ srog/) [16]. Four oyster genome files were used for the identification of SOD genes. The “cg_” and “cv_” tags were appended before the ID of these SOD genes in the published genome of *C. gigas* and *C. virginica* to distinguish these two species. Further, the IDs of *C. hongkongensis* SODs start with “CH” and those of *S. glomerata* start with “Sgl” in this study. All candidate SOD sequences with a significant BLAST hit (E-value ≤1.00 × 10^− 10^) and SOD domain (PF00080, PF00081, and PF02777) hit (E-value ≤1.00 × 10^− 10^) were obtained using the BLAST software and HMMER (http://www.ebi.ac.uk/Tools/ hmmer/search/phmmer). The presence of the SOD domain was further verified using the SMART (http://smart.embl-heidelberg.de/) web tools. The genes with partial coding sequences were filtered in the following study.

The length (aa), molecular weight (kDa), isoelectric point (pI), instability index, aliphatic index, and grand average of hydropathicity (GRAVY) values of predicted SOD molecules were calculated using the ProtParam tool (https://web.expasy.org/protparam/). Subcellular localization was predicted using softberry (http://www.softberry.com/), Euk-mPLoc 2.0 (http://www.csbio.sjtu.edu.cn/bioinf/euk-multi-2/) [55], CELLO v.2.5 (http://cello.life.nctu.edu.tw/) [56], and WoLF PSORT II (https://www.genscript.com/ wolf-psort.html) [57]. Signal peptides in the candidate genes were predicted with SignalP 5.0 server (http://www.cbs.dtu.dk/services/SignalP/) [58]. Afterward, the structures of the SOD proteins were predicted using the Phyre2 server (http://www.sbg.bio.ic.ac.uk/phyre2/html/page.cgi?id=index) [59] and SWISS-Model software (https://swissmodel.expasy.org/) [60].

### Chromosomal localization, gene duplication and Synteny analysis of SOD family members

The chromosomal positions of SODs were visualized by the TBtools software [61]. The SOD gene duplication was confirmed according to two criteria: (1) the coverage of aligned sequence was more than 70% of longer sequence, and (2) the similarity of the two aligned sequences were more than 70% [62]. Duplicated genes separated by five or fewer genes in 100-kb chromosome fragment were considered as tandem duplication genes. The synteny of SODs between four species were calculated by MCscanX method and visualized with TBtools [61].

### Multiple sequence alignments and phylogenetic analysis

The SOD protein sequences identified in the four oyster species (*C. gigas*, *C. virginica*, *C. hongkongensis*, and *S. glomerata*) and human (*Homo sapiens*), mouse (*Mus musculus*), zebrafish (*Danio rerio*), fruit fly (*Drosophila melanogaster*), and nematode (*Caenorhabditis elegans*) were retrieved from the UniProt (http://www.uniprot.org/), Ensembl (http://asia.ensembl.org/index.html), and NCBI (http://www.ncbi.nlm.nih.gov/) databases. The sequences were aligned with the MUSCLE program and a maximum-likelihood phylogenetic tree [63] with a 1000 replicate bootstrap value was constructed based on the Whelan And Goldman (WAG) model using MEGA 7.0 [64]. The conserved sequences of the candidate genes were visualized using Jalview [65]. To find conserved motifs in SOD proteins, “Multiple Em for Motif Elicitation” (MEME) version 5.3.3 (http://meme-suite.org/ tools/meme) was used to analyze protein motifs, with a maximum selection of 10 motifs for Cu/Zn SODs [66]. The conserved domains were predicted using the NCBI Batch web CD-Search Tool (https://www.ncbi.nlm.nih.gov/Structure/bwrpsb/bwrpsb.cgi) [67].

### Cis-acting elements in the promoter regions

To identify cis-regulatory elements in the SOD family genes, the 2000 bp upstream of the translation start site were retrieved from the genome of the Pacific oyster using TBtools [61]. The elements were then predicted using the AnimalTFDB 3.0 online software (http://bioinfo.life.hust.edu.cn/AnimalTFDB/) [68]. The candidate transcription factors (TFs) with q-value < 10^− 5^ were selected.

### Expression profiles of sods in developing stages and adult tissues of pacific oysters

The expression profiles of SOD genes in different developmental stages and adult tissues of Pacific oysters were analyzed using RNA-seq data [15]. The sampled developmental stages included eggs, two/four cells cell-stage embryos, early morula, morula, blastula, gastrula, trochophore, D-shape larvae, early-umbo larvae, late-term umbo larvae, pediveliger and juvenile. Collected soft tissues of adults included the mantle, gill, adductor muscle, digestive gland, hemocyte, and gonads. All samples were frozen in liquid nitrogen. The RNA-seq raw data of developing stages numbered from SRR334222 to SRR334259 and the adult tissue data numbered from SRR334212 to SRR334220 in the NCBI SRA database were downloaded. The generated RNA-seq reads were mapped to the *C. gigas* genome using Hisat2 (V2.1.0) [69]. The expression of all SOD genes was normalized and reported as fragments per kilobase of exon model per million mapped (FPKMs).

### Identification of sod gene expression patterns under abiotic and biotic stress conditions

Adult *C. gigas* were challenged with abiotic stress in a previous study [15]. The adults were acclimated in filtered and aerated seawater at 20 ± 0.5 °C for one week. The oysters were then subjected to different temperature (5, 10, 15, 20, 25, 30, 35 °C), salinity (5, 10, 15, 20, 25, 30, 40 PSU (practical salinity units), and air exposure (temperature, 20 °C; time = 1, 3, 5, 7, 9, 10, 11, days) conditions. The abiotic stress RNA-seq reads were numbered from SRR334262 to SRR334282.

For the biotic stress experiments, the acclimated oysters were injected with 100 μL of *Vibrio mediterranei* isolated from moribund *Sinonovacula constricta*, after which digestive gland tissues were sampled at 1, 3, 6, 12, 24 h post-injection. Tissue samples were frozen in liquid nitrogen and stored at − 80 °C until RNA extraction. RNA was extracted using the Trizol reagent. The extracted RNA samples were sequenced on the Illumina HiSeq 2000 platform. The expression level of all genes was quantified using Salmon v1.2.1 [70] and reported as transcripts per kilobase of exon model per million mapped reads (TPM).

### Statistical analyses

Statistical analyses were performed using the SPSS 22.0 software (IBM; NY, USA). One-way analysis of variance (ANOVA) followed by Duncan’s test was used to evaluate the expression levels of SOD genes in response to *Vibrio* stress. The results were considered significant at *P* < 0.05.

## Supplementary Information

**Additional file 1 : Table S1.** Statistic of duplicated genes in *Crassostrea gigas*, *Crassostrea hongkongensis*, and *Crassostrea virginica.*

**Additional file 2:  Fig. S1. ** synteny of SOD family members in *Crassostrea gigas*, *Crassostrea virginica, Crassostrea hongkongensis*, and *Saccostrea glomerata*. CV represented *Crassostrea virginica*; CG, *Crassostrea gigas*; CH, *Crassostrea hongkongensis*; Sgl, *Saccostrea glomerata*. The line represented the genome collinear relationship between four species. The red triangle and colored lines showed the SODs in *Crassostrea gigas* and the collinear relationship with other three species.

**Additional file 3: Table S2.** Protein pairs with high similarity between *Homo sapiens*, *Crassostrea gigas*, *Crassostrea hongkongensis*, *Crassostrea virginica*, and *Saccostrea glomerata*.

**Additional file 4: Table S3.** Sequences of Cu/Zn-SOD motifs.

**Additional file 5: Table S4.** Sub-cellular location of oyster SODs according to five databases.

**Additional file 6:Table S5.** Statistics of conserved ligand amino acids in oyster SODs.

**Additional file 7: Table S6.** Oyster SOD model predicted by phyre2.

**Additional file 8: Table S7.** Statistic of predicted protein models by SWISS-MODEL.

**Additional file 9: Table S8.** List of transcription factors and cis-element numbers of *Crassostrea gigas* SODs.

**Additional file 10: Table S9.** Full name and gene family of predicted transcription factors.

## Data Availability

The datasets of four genomes used in the current study were available in the NCBI (GCA_902806645.1, GCA_002022765.4, https://www.ncbi.nlm.nih.gov/assembly/), the China National GenBank (CNGB, https://db.cngb.org/, No. CNA0003280), as well as the Sydney rock oyster *S. glomerata* genome online database (http://soft.bioinfo-minzhao.org/srog/). The datasets used for abiotic stress analyzing were available from SRA database (PRJNA146329, https://www.ncbi.nlm.nih.gov/Traces/study/?acc=PRJNA146329&o=acc_s%3Aa). The datasets used for biotic stress analyzing during the current study available from the corresponding author on reasonable request.

## Abbreviations

ROS: Reactive oxygen species
SODs: Superoxide dismutases
H_2_O_2_: Hydrogen peroxide
CSRP: Copper-only SOD-repeat protein
pI: Isoelectric point
cyto-Cu/Zn-SOD: Cytoplasm Cu/Zn superoxide dismutase
ec-Cu/Zn-SOD: Extracellular Cu/Zn superoxide dismutase
CLs: Conserved amino acid residues that assumingly served as metal ion ligands
TFs: Transcription factors
FOX: Forkhead
AR: Androgen receptor
ESR1: Estrogen receptor 1
CNGB: China National GenBank
GRAVY: Grand average of hydropathicity
WAG: Whelan And Goldman
MEME: Multiple em for motif elicitation
FPKM: Fragments per kilobase of exon model per million mapped
TPM: Transcripts per kilobase of exon model per million mapped reads
ANOVA: Analysis of variance

## References

[1] Sies H, Berndt C, Jones DP (2017). Oxidative stress. Annu Rev Biochem.

[2] Van Raamsdonk JM, Hekimi S (2012). Superoxide dismutase is dispensable for normal animal lifespan. Proc Natl Acad Sci.

[3] Wang Y, Branicky R, Noë A, Hekimi S (2018). Superoxide dismutases: dual roles in controlling ROS damage and regulating ROS signaling. J Cell Biol.

[4] Fink RC, Scandalios JG (2002). Molecular Evolution and Structure–Function Relationships of the Superoxide Dismutase Gene Families in Angiosperms and Their Relationship to Other Eukaryotic and Prokaryotic Superoxide Dismutases. Arch Biochem Biophys.

[5] Perry JJP, Shin DS, Getzoff ED, Tainer JA (2010). The structural biochemistry of the superoxide dismutases. Bioch Biophys Proteins Proteomics.

[6] Miller A-F (2012). Superoxide dismutases: ancient enzymes and new insights. FEBS Lett.

[7] Jung I, Kim T-Y, Kim-Ha J (2011). Identification of Drosophila SOD3 and its protective role against phototoxic damage to cells. FEBS Lett.

[8] Blackney MJ, Cox R, Shepherd D, Parker JD (2014). Cloning and expression analysis of Drosophila extracellular cu Zn superoxide dismutase. Biosci Rep.

[9] Bordo D, Djinović K, Bolognesi M (1994). Conserved patterns in the Cu,Zn superoxide dismutase family. J Mol Biol.

[10] Rodríguez-Trelles F, Tarrío R, Ayala FJ (2001). Erratic overdispersion of three molecular clocks: GPDH, SOD, and XDH. Proc Natl Acad Sci U S A.

[11] Alscher RG, Erturk N, Heath LS (2002). Role of superoxide dismutases (SODs) in controlling oxidative stress in plants. J Exp Bot.

[12] Fukai T, Ushio-Fukai M (2011). Superoxide dismutases: role in redox signaling, vascular function, and diseases. Antioxid Redox Signal.

[13] Geret F, Manduzio H, Company R, Leboulenger F, Bebianno MJ, Danger JM (2004). Molecular cloning of superoxide dismutase (cu/Zn-SOD) from aquatic molluscs. Mar Environ Res.

[14] Lian S, Zhao L, Xun X, Lou J, Li M, Li X, et al. Genome-wide identification and characterization of SODs in Zhikong scallop reveals gene expansion and regulation divergence after toxic dinoflagellate exposure. Marine Drugs. 2019;17(12):700. 10.3390/md17120700 PMC6949909 31842317

[15] Zhang GF, Fang XD, Guo XM, Li L, Luo RB, Xu F, et al. The oyster genome reveals stress adaptation and complexity of shell formation. Nature. 2012;490(7418):49–54. 10.1038/nature11413 22992520

[16] Powell D, Subramanian S, Suwansa-ard S, Zhao M, O’Connor W, Raftos D, et al. The genome of the oyster Saccostrea offers insight into the environmental resilience of bivalves. DNA Res. 2018;25(6):655–65. 10.1093/dnares/dsy032 PMC6289776 30295708

[17] Robinett NG, Peterson RL, Culotta VC. Eukaryotic copper-only superoxide dismutases (SODs): a new class of SOD enzymes and SOD-like protein domains. J Biol Chem. 2018;293(13):4636–43. 10.1074/jbc.TM117.000182 PMC5880121 29259135

[18] Gonzalez M, Romestand B, Fievet J, Huvet A, Lebart MC, Gueguen Y, et al. Evidence in oyster of a plasma extracellular superoxide dismutase which binds LPS. Biochem Biophys Res Commun. 2005;338(2):1089–97. 10.1016/j.bbrc.2005.10.075 16256949

[19] Scotti PD, Dearing SC, Greenwood DR (2007). Characterisation of cavortin, the major haemolymph protein of the Pacific oyster (*Crassostrea gigas*). New Zealand J Marine Freshwater Res.

[20] Itoh N, Xue QG, Schey KL, Li Y, La Peyre JF (2011). Characterization of the major plasma protein of the eastern oyster, *Crassostrea virginica*, and a proposed role in host defense. Comp Biochem Physiol Part B Biochem Mol Biol.

[21] Holmström KM, Finkel T (2014). Cellular mechanisms and physiological consequences of redox-dependent signalling. Nat Rev Mol Cell Biol.

[22] Lackner R (1998). “Oxidative stress” in fish by environmental pollutants.

[23] Strange RW, Antonyuk S, Hough MA, Doucette PA, Rodriguez JA, Hart PJ, et al. The structure of Holo and metal-deficient wild-type human cu, Zn superoxide dismutase and its relevance to familial amyotrophic lateral sclerosis. J Mol Biol. 2003;328(4):877–91. 10.1016/s0022-2836(03)00355-3 12729761

[24] Rakhit R, Chakrabartty A (2006). Structure, folding, and misfolding of Cu,Zn superoxide dismutase in amyotrophic lateral sclerosis. Biochim Biophys Acta (BBA) Mol Basis Dis.

[25] Wood LK, Thiele DJ (2009). Transcriptional activation in yeast in response to copper deficiency involves copper-zinc superoxide dismutase. J Biol Chem.

[26] Tsang CK, Liu Y, Thomas J, Zhang Y, Zheng XF (2014). Superoxide dismutase 1 acts as a nuclear transcription factor to regulate oxidative stress resistance. Nat Commun.

[27] Regoli F, Gorbi S, Frenzilli G, Nigro M, Corsi I, Focardi S, et al. Oxidative stress in ecotoxicology: from the analysis of individual antioxidants to a more integrated approach. Mar Environ Res. 2002;54(3):419–23. 10.1016/s0141-1136(02)00146-0 12408596

[28] Andrady AL (2011). Microplastics in the marine environment. Mar Pollut Bull.

[29] Zhang H, Wang H, Chen H, Wang M, Zhou Z, Qiu L, et al. The transcriptional response of the Pacific oyster *Crassostrea gigas* under simultaneous bacterial and heat stresses. Dev Comp Immunol. 2019;94:1–10. 10.1016/j.dci.2019.01.006 30648602

[30] Gabe HB. Guerreiro AdS, Sandrini JZ. molecular and biochemical effects of the antifouling DCOIT in the mussel *Perna perna*. Comp Biochem Physiol Part C: Toxicol Pharmacol. 2021;239:108870. 10.1016/j.cbpc.2020.108870 32814145

[31] Abele D, Heise K, Pörtner HO, Puntarulo S. Temperature-dependence of mitochondrial function and production of reactive oxygen species in the intertidal mud clam *Mya arenaria*. J Exp Biol. 2002;205(13):1831–41. 10.1242/jeb.205.13.1831 12077159

[32] Abele D, Tesch C, Wencke P, Pörtner HO. How does oxidative stress relate to thermal tolerance in the Antarctic bivalve *Yoldia eightsi*? Antarct Sci. 2004;13(2):111–8.

[33] Duperthuy M, Schmitt P, Garzón E, Caro A, Rosa RD, Le Roux F, et al. Use of OmpU porins for attachment and invasion of *Crassostrea gigas* immune cells by the oyster pathogen Vibrio splendidus. Proc Natl Acad Sci. 2011;108(7):2993. 10.1073/pnas.1015326108 PMC3041119 21282662

[34] Xue Q, Beguel J-P, La Peyre J (2019). Dominin and Segon form multiprotein particles in the plasma of eastern oysters (*Crassostrea virginica*) and are likely involved in Shell formation. Front Physiol.

[35] Doonan R, McElwee JJ, Matthijssens F, Walker GA, Houthoofd K, Back P, et al. Against the oxidative damage theory of aging: superoxide dismutases protect against oxidative stress but have little or no effect on life span in *Caenorhabditis elegans*. Genes Dev. 2008;22(23):3236–41. 10.1101/gad.504808 PMC2600764 19056880

[36] Landis GN, Tower J (2005). Superoxide dismutase evolution and life span regulation. Mech Ageing Dev.

[37] Bunting K, Cooper JB, Badasso MO, Tickle IJ, Newton M, Wood SP, et al. Engineering a change in metal-ion specificity of the iron-dependent superoxide dismutase from *Mycobacterium tuberculosis*-- X-ray structure analysis of site-directed mutants. Eur J Biochem. 1998;251(3):795–803. 10.1046/j.1432-1327.1998.2510795.x 9490054

[38] Benzie IFF (2000). Evolution of antioxidant defence mechanisms. Eur J Nutr.

[39] Meng J, Wang W-X, Li L, Zhang G (2021). Accumulation of different metals in oyster *Crassostrea gigas*: significance and specificity of SLC39A (ZIP) and SLC30A (ZnT) gene families and polymorphism variation. Environ Pollut.

[40] Gleason JE, Galaleldeen A, Peterson RL, Taylor AB, Holloway SP, Waninger-Saroni J, et al. Candida albicans SOD5 represents the prototype of an unprecedented class of cu-only superoxide dismutases required for pathogen defense. Proc Natl Acad Sci U S A. 2014;111(16):5866. 10.1073/pnas.1400137111 PMC4000858 24711423

[41] Strange RW, Antonyuk SV, Hough MA, Doucette PA, Valentine JS, Hasnain SS (2006). Variable metallation of human superoxide dismutase: atomic resolution crystal structures of cu-Zn, Zn-Zn and as-isolated wild-type enzymes. J Mol Biol.

[42] Ruan Z, Liu Y, Chang G, Lin Z, Xue Q (2022). Molecular characterization of two CuZn-SOD family proteins in the Pacific oyster *Crassostrea gigas*. Comp Biochem Physiol B: Biochem Mol Biol.

[43] Xue Q, Gauthier J, Schey K, Li Y, Cooper R, Anderson R, et al. Identification of a novel metal binding protein, segon, in plasma of the eastern oyster, *Crassostrea virginica*. Comp Biochem Physiol Part B Biochem Mol Biol. 2012;163(1):74–85. 10.1016/j.cbpb.2012.05.002 22580268

[44] Crow JP, Sampson JB, Zhuang Y, Thompson JA, Beckman JS (1997). Decreased zinc affinity of amyotrophic lateral sclerosis-associated superoxide dismutase mutants leads to enhanced catalysis of tyrosine nitration by Peroxynitrite. J Neurochem.

[45] Lamb AL, Torres AS, O'Halloran TV, Rosenzweig AC (2001). Heterodimeric structure of superoxide dismutase in complex with its metallochaperone. Nat Struct Biol.

[46] Waninger-saroni J, Gleason J, Galaleldeen A, Hart P, Culotta V. The crystal structure of SOD5: an unusual copper-only superoxide dismutase (774.2). FASEB J. 2014;28(S1):774.2.

[47] Davey RA, Grossmann M (2016). Androgen receptor structure, function and biology: from bench to bedside. Clin Biochem Rev.

[48] Schwabe JWR, Teichmann SA (2004). Nuclear receptors: the evolution of diversity. Sci STKE.

[49] Qi H, Li L, Zhang G. Construction of a chromosome-level genome and variation map for the Pacific oyster *Crassostrea gigas*. Mol Ecol Resour. 2021;21(5):1670–85. 10.1111/1755-0998.13368 33655634

[50] Haberer G, Hindemitt T, Meyers BC, Mayer KFX (2004). Transcriptional similarities, dissimilarities, and conservation of cis-elements in duplicated genes of Arabidopsis. Plant Physiol.

[51] Lynch M, Conery JS (2000). The evolutionary fate and consequences of duplicate genes. Science (New York, NY).

[52] Duveau F, Yuan DC, Metzger BPH, Hodgins-Davis A, Wittkopp PJ. Effects of mutation and selection on plasticity of a promoter activity in *Saccharomyces cerevisiae*. Proc Natl Acad Sci. 2017;114(52):E11218. 10.1073/pnas.1713960115 PMC5748197 29259117

[53] Peñaloza C, Gutierrez AP, Eöry L, Wang S, Guo X, Archibald AL, et al. A chromosome-level genome assembly for the Pacific oyster *Crassostrea gigas*. GigaScience. 2021;10:3. 10.1093/gigascience/giab020 PMC7992393 33764468

[54] Peng J, Li Q, Xu L, Wei P, He P, Zhang X, et al. Chromosome-level analysis of the *Crassostrea hongkongensis *genome reveals extensive duplication of immune-related genes in bivalves. Mol Ecol Resour. 2020;20(4):980–94. 10.1111/1755-0998.13157 32198971

[55] Chou K-C, Shen H-B (2010). A new method for predicting the subcellular localization of eukaryotic proteins with both single and multiple sites: Euk-mPLoc 2.0. PLoS One.

[56] Yu C-S, Chen Y-C, Lu C-H, Hwang J-K (2006). Prediction of protein subcellular localization. Proteins: Struct Funct Bioinform.

[57] Horton P, Park K-J, Obayashi T, Fujita N, Harada H, Adams-Collier CJ, et al. WoLF PSORT: protein localization predictor. Nucleic Acids Res. 2007;35(Web Server issue):W585–7. 10.1093/nar/gkm259 PMC1933216 17517783

[58] Nielsen H, Tsirigos KD, Brunak S, von Heijne G (2019). A brief history of protein sorting prediction. Protein J.

[59] Kelley LA, Mezulis S, Yates CM, Wass MN, Sternberg MJE (2015). The Phyre2 web portal for protein modeling, prediction and analysis. Nat Protoc.

[60] Waterhouse A, Bertoni M, Bienert S, Studer G, Tauriello G, Gumienny R, et al. SWISS-MODEL: homology modelling of protein structures and complexes. Nucleic Acids Res. 2018;46(W1):W296–303. 10.1093/nar/gky427 PMC6030848 29788355

[61] Chen C, Chen H, Zhang Y, Thomas HR, Frank MH, He Y, et al. TBtools: an integrative toolkit developed for interactive analyses of big biological data. Mol Plant. 2020;13(8):1194–202. 10.1016/j.molp.2020.06.009 32585190

[62] Zhao P, Wang D, Wang R, Kong N, Zhang C, Yang C, et al. Genome-wide analysis of the potato Hsp20 gene family: identification, genomic organization and expression profiles in response to heat stress. BMC Genomics. 2018;19(1):61. 10.1186/s12864-018-4443-1 PMC5774091 29347912

[63] Guindon S, Gascuel O (2003). A simple, fast, and accurate algorithm to estimate large phylogenies by maximum likelihood. Syst Biol.

[64] Kumar S, Stecher G, Tamura K (2016). MEGA7: molecular evolutionary genetics analysis version 7.0 for bigger datasets. Mol Biol Evol.

[65] Waterhouse AM, Procter JB, Martin DMA, Clamp M, Barton GJ (2009). Jalview version 2—a multiple sequence alignment editor and analysis workbench. Bioinformatics.

[66] Bailey TL, Boden M, Buske FA, Frith M, Grant CE, Clementi L, et al. MEME suite: tools for motif discovery and searching. Nucleic Acids Res. 2009;37(suppl_2):W202–8. 10.1093/nar/gkp335 PMC2703892 19458158

[67] Lu S, Wang J, Chitsaz F, Derbyshire MK, Geer RC, Gonzales NR, et al. CDD/SPARCLE: the conserved domain database in 2020. Nucleic Acids Res. 2020;48(D1):D265–d268. 10.1093/nar/gkz991 PMC6943070 31777944

[68] Hu H, Miao Y-R, Jia L-H, Yu Q-Y, Zhang Q, Guo A-Y (2019). AnimalTFDB 3.0: a comprehensive resource for annotation and prediction of animal transcription factors. Nucleic Acids Res.

[69] Kim D, Langmead B, Salzberg SL (2015). HISAT: a fast spliced aligner with low memory requirements. Nat Methods.

[70] Patro R, Duggal G, Love MI, Irizarry RA, Kingsford C (2017). Salmon provides fast and bias-aware quantification of transcript expression. Nat Methods.

