# Supplementary material for: Genome-wide identification and characterization of superoxide dismutases in four oyster species reveals functional differentiation in response to biotic and abiotic stress
Source: BMC Genomics. 2022 May 18;23:378. doi: 10.1186/s12864-022-08610-9 (PMC9118643; doi:10.1186/s12864-022-08610-9)
Supplement: Supplementary file 2 — Additional file 2: Fig. S1. synteny of SOD family members in Crassostrea gigas, Crassostrea virginica, Crassostrea hongkongensis, and Saccostrea glomerata. CV represented Crassostrea virginica; CG, Crassostrea gigas; CH, Crassostrea hongkongensis; Sgl, Saccostrea glomerata. The line represented the genome collinear relationship between four species. The red triangle and colored lines showed the SODs in Crassostrea gigas and the collinear relationship with other three species. [file 12864_2022_8610_MOESM2_ESM.docx]

**Fig S1** synteny of SOD family members in *Crassostrea gigas*, *Crassostrea virginica, Crassostrea hongkongensis*, and *Saccostrea glomerata*.

CV represented *Crassostrea virginica*; CG, *Crassostrea gigas*; CH, *Crassostrea hongkongensis*; Sgl, *Saccostrea glomerata*. The line represented the genome collinear relationship between four species. The red triangle and colored lines showed the SODs in *Crassostrea gigas* and the collinear relationship with other three species.
