# Supplementary material for: Genome-wide identification and characterization of superoxide dismutases in four oyster species reveals functional differentiation in response to biotic and abiotic stress
Source: BMC Genomics. 2022 May 18;23:378. doi: 10.1186/s12864-022-08610-9 (PMC9118643; doi:10.1186/s12864-022-08610-9)
Supplement: Supplementary file 4 — Additional file 4: Table S3. Sequences of Cu/Zn-SOD motifs. [file 12864_2022_8610_MOESM4_ESM.docx]

**Table S3.** Sequences of Cu/Zn-SOD motifs

| Motif | logo | sequences | Width | Motif Similarity Matrix | | | | | | | | |  |
| --- | --- | --- | --- | --- | --- | --- | --- | --- | --- | --- | --- | --- | --- |
|  |  |  |  | 1 | 2 | 3 | 4 | 5 | 6 | 7 | 8 | 9 | 10 |
| 1 | 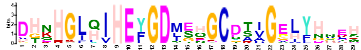 | DHNHGLQIHEYGDMEHGCDTIGELYHNEH | 29 | - |  |  |  |  |  |  |  |  |  |
| 2 | 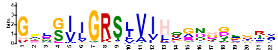 | GPLGIJGRSJVIHQGBHGSGRV | 22 | 0.17 | - |  |  |  |  |  |  |  |  |
| 3 | 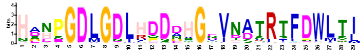 | HANPGDLGDLHDDDHGEVNATRTFDWLTI | 29 | 0.18 | 0.17 | - |  |  |  |  |  |  |  |
| 4 | 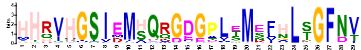 | HHRVHGSIEMHQRGDGPLEMEFHLSGFNV | 29 | 0.13 | 0.19 | 0.22 | - |  |  |  |  |  |  |
| 5 | 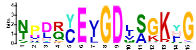 | NPDRYEVGDLSGKYG | 15 | 0.33 | 0.15 | 0.24 | 0.16 | - |  |  |  |  |  |
| 6 | 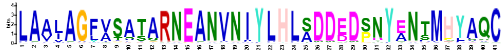 | LAALAGFVSATARNEANVNIYLHLSDDEDSNYENTMHYAQC | 41 | 0.16 | 0.16 | 0.18 | 0.24 | 0.18 | - |  |  |  |  |
| 7 | 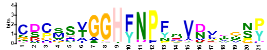 | CDCSSVGGHFNPFNVBVEGNP | 21 | 0.3 | 0.18 | 0.24 | 0.22 | 0.23 | 0.13 | - |  |  |  |
| 8 | 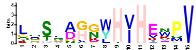 | LQSLAGGWHVHENPV | 15 | 0.29 | 0.2 | 0.12 | 0.18 | 0.1 | 0.23 | 0.25 | - |  |  |
| 9 | 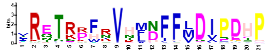 | VRETRRFRVHMBFFLDIPDHP | 21 | 0.22 | 0.14 | 0.18 | 0.23 | 0.16 | 0.39 | 0.17 | 0.27 | - |  |
| 10 | 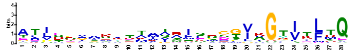 | ATJEPVVKVTTATRISRGGVKGTITLTQ | 28 | 0.08 | 0.19 | 0.11 | 0.24 | 0.15 | 0.21 | 0.12 | 0.16 | 0.19 | - |
